# Postseparation Financial Abuse Perpetrated Through Government Systems: A Survey of Australian Mothers’ Experiences of Child Support

**DOI:** 10.1177/10778012241280057

**Published:** 2024-09-19

**Authors:** Kay Cook, Adrienne Byrt, Terese Edwards, Rachael Burgin

**Affiliations:** 1 Centre for Transformative Media Technologies, 3783 Swinburne University of Technology, Hawthorn, Victoria, Australia; 2 Single Mother Families Australia, Adelaide, Australia; 3 Swinburne Law School, 3783 Swinburne University of Technology, Hawthorn, Victoria, Australia

**Keywords:** child support, financial abuse, postseparation, single mothers, systems abuse

## Abstract

Financial abuse is a form of violence that can extend well beyond intimate partner relationship breakdown. A survey of 540 Australian separated parents examined how financial abuse was perpetrated, with a focus on child support and other government systems. Women reported that their ex-partners minimized and withheld child support payments to inflict direct financial harm, while interactions between the family court, taxation, and benefit payment systems were taken advantage of to threaten or control them. Findings revealed how the mandatory and complex Australian child support system provided perpetrators with a useful means of perpetrating financial abuse across households.

## Introduction

Recent Australian research contends that women living with violent partners face a choice between staying with violence or leaving in poverty (Summers, 2022). Women's purported “choice” references the inadequate rate of financial support available to single-parent families that renders them Australia's most impoverished family type (Davidson et al., 2020). However, in this analysis, we contend that even after leaving violent partners, single mothers face ongoing abuse, albeit in a form that foregrounds financial over physical violence. Further, this violence is facilitated by the state (Natalier, 2018) as the government requires separated mothers to engage with the child support system, which renders the value of women's government-provided family payments dependent upon and open to manipulation by their abusive ex-partners.

We contend that the Australian child support system, like others operating elsewhere, can be an abusive system for both mothers and fathers with limited financial means (Cook et al., 2023; Cozzolino & Williams, 2017; Natalier, 2012, 2018), whereby fathers’ inability to pay can result in penalties while women's and children's basic needs go unmet. In particular, we focus on two features of the Australian child support system that make payment discrepancies difficult to detect, while also multiplying the financial ramifications for payees in some cases. These features include (a) the inclusion of child support as income in the calculation of family payments, which reduces recipients’ family payments by 50 cents for every dollar of child support received; and (b) the use of the “expected” rather than the “received” amount of child support to calculate family payment entitlements when child support is received privately rather than via the Department, which is the case for half of the caseload (DSS, 2023). As will be detailed shortly, these provisions produce an incentive for abusive ex-partners to agree to­—but not pay—high-value, child support orders. Where private child support is not paid, recipient parents not only experience a shortfall in their expected child support income but also lose almost half this value again in the form of reduced family payments. In addition, when payers lodge late tax returns, even years after the fact, the “expected” amount of child support can be retrospectively calculated and regarded as “received.” As a result, payees can be subject to retrospectively applied family payment debts, which are owed to the state and vigorously pursued.

The harms perpetrated against low-income families by the system result from the privatization of financial dependency, where financial responsibility for children's welfare is shifted away from the state and onto separated parents (Diduck, 1995; Eekelaar, 1991a), a practice that has been regarded as morally flawed (Cook, 2019; Eekelaar, 1991b). An extreme example of the use of state debts to discipline ‘problematic populations’ can be seen in the United States, where it is disproportionately poor black fathers who have faced jail time for failing to meet unrealistically high child support liabilities (Cozzolino, 2018; Cozzolino & Williams, 2017; Rush, 2015).

While the child support system itself can inflict harm upon both mothers and fathers, particularly in low-income families, here we examine how the system can be intentionally exploited by malicious ex-partners, compounding the harms experienced by already vulnerable low-income women. While both parents experience a reduced financial capacity and economy of scale following separation, the financial costs of separation are most keenly and enduringly felt by single mothers (de Vaus et al., 2017), whose disproportionate responsibility for children's care renders them financially dependent on their ex-partner: a responsibility enforced by the state. Given these mandated relations of private dependence, we contend that the Australian system compounds child support's inherent potential for harm in gendered ways, by affording family violence perpetrators specific opportunities to wield state-sanctioned debts as weapons against their impoverished ex-partners. International and Australian research has implicated child support in financially abusive postseparation relationships (Kaittila et al., 2022; Natalier, 2018; Sharp-Jeffs, 2021; Tegler et al., 2023; Women's Legal Services Australia, 2024). However, despite advances in measuring economic abuse (Postmus et al., 2016), research has yet to quantify the nature of financial abuse experienced by separated mothers, or how child support is implicated in its perpetration. In response, and in light of the release of the *Australian National Plan to End Violence Against Women and Children* [National Plan] (DSS, 2022b) that lists financial abuse as a priority issue, we draw on survey data to explore women's experiences of these issues.

To examine the compounding experience of harm experienced by low-income women with abusive ex-partners, we conducted the first quantitative study of Australian study of women's experiences of child-support-perpetrated financial abuse. Our exploratory study sought to answer the following research questions:

1. To what extent did Australian mothers experience financial abuse postseparation?
2. How did separated mothers experience financial abuse perpetrated through the child support system?
3. How did financially abused mothers’ child support payments differ from those who did not report abuse?

By drawing on the lived experiences of victim-survivors, this study identifies how the Australian child support system can be intentionally misused to enact financial abuse, and more importantly, identifies ways that these tactics can be disrupted. Our findings, which center women's experiences, can inform victim-centered policy and practical interventions to reduce the incidence and impact of abuse achieved through the state imposition of debt, as well as identifying where further research is needed. Such insights have not been possible to derive from existing administrative or other panel study data.

We begin our investigation by describing the nature of postseparation financial abuse and the features of the Australian child support system that make such abuse possible. We then introduce our approach to survey design and analysis, before presenting our findings in response to our three research questions. We conclude with a discussion of how findings resonate with or deviate from previous financial abuse and child support research, and where there are opportunities for future research and reform to address issues arising from previously unquestioned policy assumptions.

## Postseparation Financial Abuse

Economic abuse is an umbrella term that includes financial abuse alongside other forms of economic harm, such as employment sabotage and the restriction or control of resources such as housing and transportation, as perpetrators work to control a victim's economic autonomy (Johnson et al., 2022; Postmus et al., 2012, 2016, 2020; Sharp-Jeffs, 2021). Financial abuse is a subtype of economic abuse, whereby perpetrators control money and finances, including their (ex-)partner's access to these resources. Governments (DSS, 2022b) and researchers (PenzeyMoog & Slakoff, 2021, p.645; Sharp-Jeffs, 2021) have described financial abuse as “among the most powerful methods abusers have to keep a survivor in a relationship and to diminish their ability to safely leave.” Research suggests that financial abuse is prevalent within violent intimate partner relationships, with rates ranging from 38% to 98% (Boxall & Morgan, 2021; Postmus et al., 2016). However, while financial abuse is implicated in keeping women in abusive relationships, the extent to which financial abuse continues after separation is largely unknown.

Given the ubiquity of financial technologies in everyday life, financial abuse often involves the use of third-party systems, whereby banking, credit, and insurance systems may be manipulated by perpetrators to enact harm (Cook et al., 2023; Fitzpatrick, 2022; PenzeyMoog & Slakoff, 2021). In Australia, the systems implicated in the perpetuation of financial abuse include coercing victim-survivors into taking out loans; preventing the withdrawal of funds from banking or investment accounts; avoiding loan repayments; refusing or delaying selling a jointly owned home; and failing to abide by court orders regarding property or financial settlements (Douglas, 2021; Kaye et al., 2021). What little empirical research has been conducted on how financial abuse is perpetrated through third-party systems has, however, extended the purview beyond the financial and legal sectors (Chaudhri, 2020; Natalier, 2018). Research has examined how government income and payment systems, such as child support, welfare benefits, and taxation systems, can also be manipulated by perpetrators to inflict financial harm on victim-survivors (Douglas & Nagesh, 2019; Kaittila et al., 2022; Natalier, 2018; Shephard, 2005; Tegler et al., 2023). Indeed, when intimate partner relationships end, some means of perpetrating financial harm become unavailable. Here, the mandatory continuation of financial ties across households, such as is required for Australian family payment recipients through child support, can make government systems a useful tool in perpetrators’ enactment of ongoing control and violence.

### Intersections Between Relationship Surveillance, State Debts, and Postseparation Financial Abuse

The opportunities for Australian policy to be used to inflict financial harm upon welfare recipients, primarily single mothers, are evident in a range of policy requirements. Most notoriously, the Australian Government faced significant criticism of what was deemed to be an illegal “Robodebt” scheme, whereby automatically generated and erroneous debts were raised against income support recipients without any legal basis (Rinta-Kahila et al., 2023; Whiteford, 2021). However, while this debt scheme was abandoned, legislation to apply debts to benefit recipients has been central to the Australian system. More insidiously, and unlike the Robodebt scheme that was applied to a wide range of recipients, numerous policies exist that levy government debts on the basis of claimants’ (postseparation) relationship status and behavior.

Previously, benefit recipients have been required to have a witness verify their relationship status, to “catch” people flouting the rules (Knaus, 2018) by declaring they are single when in fact they have a partner whose finances they can draw on. While only 1.3% of the 75,000 welfare recipients subjected to the review were “caught” between January 2018 and November 2019 (Henriques-Gomes, 2021), what was not acknowledged in government or media reporting was the possibility for malicious ex-partners to instigate or manipulate these reviews to impose debts on separated parents whose relationships were found to be outside of the social security rules.

More recently, the 2023–24 federal budget contained changes to decision-making processes that require a consideration of family and domestic violence when assessing whether a person is a member of a couple for social security purposes. Previously, in making determinations, the “couple rule” was applied under the Social Security Act, 1991 (Cth) section 4(3) (Sleep, 2019)). If a family violence victim survivor was assessed as being a member of a couple, both their and the perpetrator's income and assets would be evaluated jointly, which could lead to victim-survivors being denied income support payments or result in benefit overpayment debts and even criminal prosecutions for social security fraud if the stated relationship was later declared inaccurate (Sleep, 2019).

Finally, and most relevant to the present study, to receive government family payments, called Family Tax Benefits (FTB), separated parents in Australia, who are overwhelmingly mothers (Department of Social Services [DSS], 2022a), are required to seek child support from their ex-partner. Child support reduces the value of FTB payments by approximately 50c for every dollar of support received (Skinner et al., 2017) and can result in recipients owing FTB debts to the state where child support payments are not accurately declared. However, payers can manipulate their child support liabilities and payments which may result in payee FTB debts raised against their ex-partners. In extreme cases, such as when lump-sum child support payments are received by private recipients, FTB debts can be raised against child support recipients and vigorously pursued by the government. It is the dependence of FTB payments on child support income, alongside Australia's problematic encouragement of private payment arrangements (Australian Government, 2023; HRSCSPLA, 2015) that makes child support particularly useful in the perpetration of postseparation financial abuse. However, given their private nature, there is very little data available on how financial abuse is perpetuated. As such, it was women's experience of financial abuse perpetrated through child support that this study sought to examine.

As these examples illustrate, Australian social security legislation provides significant avenues for the intentional manipulation of relationship governance mechanisms and benefit payment and debt processes to inflict harm on an ex-partner. However, while government systems are often implicated, government review processes have only recently begun to identify specific mechanisms through which financial harm is perpetrated.

Financial abuse is directly noted within the Australian Government's National Plan (DSS, 2022b), where refusing to pay back loans or sending abusive messages through bank transfers are recognized among a suite of behaviors that primarily target women. Similarly, a Parliamentary Inquiry into Family, Domestic and Sexual Violence (House of Representatives Standing Committee on Social Policy and Legal Affairs [HRSCSPLA], 2021), the 2023 Federal Women's Budget Statement (Australian Government, 2023) and a Joint Select Committee on Australia's Family Law System (JSCAFLS) (2021a, 2021b), each describe financial abuse occurring within institutional systems. On these issues, an independent Interim Economic Inclusion Advisory Committee (2023, p.78) report published by the government notes that the relationship between FTB payments and child support “provides a means for malicious ex-partners to inflict financial abuse.” The JCAFLS recommended that the child support system, overseen by the government department, Services Australia, acknowledges child support underpayment and nonpayment as indicators of abuse, and called for the training for Services Australia staff to recognize family violence in child support cases. Similarly, the National Plan (DSS, 2022b) encourages institutions to build their capacity to prevent and respond to financial abuse, abuse of process, and technology-facilitated violence, however, little research exists to guide such reform. While multiple government inquiries and reports highlight the role that child support may play in the perpetration of financial abuse, further evidence is required to identify how financial abuse is perpetrated and what specific reforms would effectively intervene.

### Features of the Australian Child Support Scheme That Afford Financial Abuse

As was described earlier, it is the contingency of FTB on child support that exposes women to the risk of financial harm, as child support becomes a useful tool for malicious ex-partners to exert financial control long after relationships have ended. However, despite 85% of separated parents receiving FTB (DSS, 2022a), very little is known about mothers’ experiences of postseparation financial abuse perpetrated through government systems. Here, we describe the process through which parents calculate and transfer child support to identify where the system affords opportunities for financial abuse.

The amount of child support that parents can expect to receive is calculated using a “basic” eight step formula (Services Australia, 2023). The child support formula equally considers each parents’ proportion of combined income and share of overnight care of children. The “equal treatment” of both parents means that no special consideration is given to women's lower earning capacity due to the gender wage gap, or disproportionate likelihood of having care of children during the working week, compared to separated fathers’ normative caring pattern of “every second weekend and half that school holidays,” which is less likely to interfere with their earning capacity (Cook, 2019). As a result, each dollar of income and each night of children's overnight care can become a site of contest and potential weaponization, with harms disproportionately impacting on low-income women.

Numerous reviews of the child support scheme have found that parents – primarily fathers who are less reliant on government forms of income, and thus are subject to less government scrutiny – can manipulate their child support liabilities by lowering their taxable incomes (Australian Law Reform Commission, 2012; House of Representatives Standing Committee on Family and Community Affairs, 2003; House of Representatives Standing Committee on Social Policy and Legal Affairs, 2015). This can be achieved through legitimate or illegitimate means. These government reviews and additional academic inquiries (Cook, 2013; Shephard, 2005) have identified such methods as working cash in hand, failing to lodge tax returns or lodging late returns, and self-employed parents diverting income to a business, a trust, or a new partner. At the same time, parents can also manipulate their share of care time. This can be achieved by one parent registering a parenting order with child support that stipulates more care time than they actually take up. Such discrepancies may exist when court-ordered or mutually determined parenting agreements are registered but not abided by or are changed unilaterally. Research has suggested that the costly, administratively onerous, legally fraught, relationally challenging, and/or physically dangerous nature of attempts to reach more accurate agreements through the courts, mediators, the government “Change of Assessment” process or through personal negotiations may prevent mothers from resolving care-time discrepancies within the child support formula (Cook et al., 2023; Douglas & Nagesh, 2019).

Finally, regardless of what income and care-time arrangements are entered into the formula, the resultant child support order can also offer opportunities for financial abuse. Perpetrating parents may manipulate what payments are made and the timing of these payments, with reports suggesting that perpetrators skip payments immediately before Christmas, children's birthdays, or the start of the school year to maximize the financial and psychological harms inflicted on low-income mothers (Cook et al., 2023). Here, Australian quantitative research has shown that when less child support was received in the previous month than was expected, children experienced significantly lower “educational well-being” than those whose mothers received the expected payment (Cook et al., 2008). Such withholding by abusive ex-partners may be designed to limit mothers’ attempts to show care or facilitate their children's social inclusion through their use of child support money, and buttresses payers’ desire to control how “*their*” money is spent (Cook & Skinner, 2021; Natalier & Hewitt, 2010). Taken together, research suggests that it is the value, timing, and predictability of payments that are significant to low-income single-parent family well-being, and thus can be manipulated by perpetrators to induce harm.

## Methods

### Collaboration and Survey Design

In collaboration with the National Council of Single Mothers and their Children (NCSMC), now known as Single Mother Families Australia, an anonymous online survey was developed to examine separated parents’ postseparation finances. The survey captured women's experiences of postseparation financial safety, including how this safety could be jeopardized by financial abuse perpetrated through systems such as child support, tax, and government benefit systems. Where appropriate, demographic, benefit payment, and child support survey questions were aligned with those used in Australian panel studies to allow for comparisons with nationally representative data. While it is an important tool, the Scale of Economic Abuse (Postmus et al., 2016) was not readily applicable to systems-perpetrated financial abuse, particularly in a postseparation context, and so was not included in the survey.

### Ethical Considerations

A draft version of the survey was piloted with 10 members of the NCSMC advisory board, all single mothers, to ensure the appropriateness of the survey questions and wording. Ethics approval was then obtained from [University removed for blinding] (Application ID: 20226091-11094) before the survey's distribution. The psychological safety of participants who may have experienced current or previous family violence was prioritized by allowing participants to not answer each question, to skip ahead to the next survey section at any point within the block of questions pertaining to family violence, and also to exit the survey at any point. After each family violence question, the contact details of support organizations were also provided.

### Recruitment and Data Collection

The survey was promoted through the NCSMC's social media platforms, with the researchers and other advocacy and social welfare organizations also sharing this call for participants within their networks. Administered using the Qualtrics online data collection platform from 1 October 2022 to 31 January 2023, the survey attracted 540 respondents. Of these, 23 respondents (4%) were exited after the first question, as they answered “no” to being a single, sole, or repartnered parent with a dependent child under 18 years of age. Email feedback received from some such respondents indicated that this group included mothers whose ex-partner had obtained sole care of their child (ren), which these mothers—and researchers (Vnuk, 2019)—regarded as an extreme example of legal and financial abuse. This exclusion illustrates a limitation of the current study, which failed to sufficiently examine the nexus between legal and financial abuse (Scott, 2023) by excluding mothers without a resident dependent child.

Respondents were asked how much child support they expected to receive in the previous month, with a follow-up question asking how much they actually received. Respondents could enter a free text number or comment, or select “don’t know.” Data were cleaned and converted into a dollar value for each question, with two extreme outliers that greatly exceeded child support payment amounts (e.g., $16,000 child support expected in the previous month) removed.

### Data Analysis

Data were imported from Qualtrics into SPSS for analysis. Chi quare tests were conducted to assess the distribution of the sample across discrete analytical groups, while independent sample t-tests were used to compare the distribution of continuous data between groups.

## Results

### Sample Characteristics

Almost all (99.1%) respondents identified as female (Table 1). As such, when reporting our results, we refer to participants as “mothers” or “women.” This is not to diminish the experiences of single fathers (*n* = 1) or gender-diverse participants (*n* = 3) but to reflect the gender of the overwhelming majority of our participants. Again, we acknowledge that for this small group of respondents, “merging or omitting” (Vnuk, 2010) their data erases their unique experiences. Future surveys dedicated to the experiences of single fathers and gender-diverse parents are recommended.

**Table 1.** Participant Demographics.

	N	%
Gender		
Female	462	99.1
Male	1	0.2
Nonbinary	2	0.4
Other	1	0.2
Age	445	*M* = 44.78
	*SD* = 6.75, Range: 28–61	
Age of youngest child living with you	441	*M* = 10.43
	*SD* = 4.46, Range: 1–24	
Number of children		
1	139	29.8
2	160	34.3
3	87	18.6
4	50	10.7
5 or more	31	6.6
Relationship to study child		
Adoptive parent	1	0.2
Biological parent	459	98.3
Foster parent	2	0.4
Guardian	2	0.4
Step parent	1	0.2
Other	2	0.4
Aboriginal or Torres Strait Islander		
Yes	21	4.5
No	446	95.5
Study child is Aboriginal or Torres Strait Islander		
Yes	27	5.8
No	439	94.2
Born in Australia		
Yes	402	86.1
No	65	13.9
Primary language spoken at home		
English	459	98.9
Spanish	2	0.4
Greek	1	0.2
Other	2	0.4
Highest level of education completed		
Some high school	20	4.3
Completed high school	29	6.3
TAFE, trade certificate, or diploma	169	36.6
University, CAE, or some tertiary institute degree	124	26.8
Postgraduate qualification	109	23.6
Location of residence		
City	279	60.3
Regional town	139	30.0
Rural area	41	8.9
Remote community	2	0.4
Did you do any work in a job last week?		
Yes, worked in one job	257	55.5
Yes, worked in more than one job	47	10.2
No	159	34.3
Current labor force status		
Employed full time	120	25.9
Employed part time	122	26.3
Casually employed	67	14.4
Studying	44	9.5
Looking for work	39	8.5
Not working and not looking for work	50	10.8
Main source of income		
Commission	6	1.3
Government pension, allowance or payment	184	40.9
Wages or salary	260	57.8
Usual total weekly income after tax		
Negative income or zero	21	4.8
$1–$379	37	8.5
$380–$769	145	33.4
$770–$1149	110	25.4
$1150–$1919	79	18.2
$1920 or more	29	6.7
Receipt of government income support payments		
Yes	369	81.1
No, Australian citizen	83	18.2
No, noncitizen who is not eligible for payments	3	0.7
Government payment that you most rely on		
Carer allowance	13	3.0
Carer payment	45	10.5
Disability support pension	17	4.0
Family tax benefit only (no other benefits)	99	23.1
JobSeeker	66	15.4
Parenting payment partnered	5	1.2
Parenting payment single	110	25.6
Youth allowance (other/unemployed)	1	0.2
Youth allowance (student)	1	0.2
Other	15	3.5
None of these	50	11.7
Years in receipt of a government payment		
Less than 6 months	8	2.2
6 months to 11 months	13	3.5
1 year to 5 years	143	38.8
More than 5 years	190	51.5
Not sure	5	1.4
Prefer not to say	10	2.7
How do you receive child support payments?		
Child support agency (agency collect)	233	66.8
Directly from the other parent (private collect)	49	14.0
Neither	56	16.0
Don’t know	7	2.3
Prefer not to say	4	1.1

The *M*_age_ of respondents was 45 and most (64.1%) respondents had two or fewer children. The mean age of respondents’ youngest child living at home was 10 years. Such single parents—with a youngest child over age 8 in receipt of government income support payments—would have been subject to “mutual obligations” at the time of the survey, requiring them to seek 30 h of employment per fortnight as a condition of benefit eligibility. The most common total weekly after-tax income band was between $380 and $769 (approximately US$250 and $506), with 33.4% of the sample reporting such an income. This is well below the (pre-tax) 2022 Australian female average weekly earnings for all female employees of $1,144.30 (approximately US$753) (ABS, 2022), excluding supplemental government payments, such as FTB. While Australia has never adopted an official poverty line (Interim Economic Inclusion Advisory Committee, 2023), reports of poverty based on weekly income, including housing costs, for a working single parent with two children is $958.16 (approximately US$631) (Tejani, 2022). As such, in line with research that identifies single-mother families as Australia's most impoverished family type (Davidson et al., 2020), our survey respondents appeared to be more financially vulnerable than the general population, making financial abuse particularly threatening to their family's financial well-being. Overall, 81.1% of respondents reported receiving some form of government payment. This is consistent with reports that 85% of families receive the generously means-tested family payments (DSS, 2022a).

#### Child Support Characteristics

In terms of how mothers collected child support, two thirds (66.8%) collected payments via the government agency, Services Australia. While 50% of the Australian caseload transfers payments privately (DSS, 2022a), only 14.2% of women in our sample did so. Given that government data reveals that over two thirds of women commence by collecting child support privately (Oldham & Smyth, 2018), participants may have switched to Agency Collect after experiencing compliance issues; however, this is unknown. A slightly larger proportion of women than those who were collecting privately reported either that they did not know how they received payments or that they received them through neither method (18.1%).

As our survey asked how women *received* payments, and not how women *should* receive payments, it could be that payments for these women were never or were only rarely received. If so, it is likely that these women were also registered to receive payments privately, as otherwise they would have been provided with monthly payment statements by Services Australia indicating how much money they were due to receive. However, again, this is speculative. A limitation created by the low number of women who indicated receiving private payments was that we were unable to use their collection method as a grouping variable in our analyses, despite it playing a role in modifying women's experience of FTB calculations.

### The Extent of Postseparation Financial Abuse

Almost nine in 10 (87.6%) women reported having ever felt that someone else was controlling their money or finances (Table 2). Of those women who reported feeling financially controlled, almost all women (95.1%) reported that control was exerted by their ex-partner, with others indicating control by their current partner. Of those women who reported financial control by their ex-partner, most (58.8%) reported that this control was still occurring. For women who reported financial control by their ex-partner, the behavior had most commonly occurred for more than 10 years (43.6%), with another third of women (32.3%) experiencing such control for between 5 and 10 years.

**Table 2.** Financial Abuse and Child Support Characteristics.

	N	%
Have you ever felt someone else has control over your money or finances?		
Yes	376	87.6
No	53	12.4
If yes, this control was by:		
Ex-partner	353	95.1
Current partner (married or de facto)	8	2.2
Teenage or adult child	1	0.3
Another family member or friend	4	1.1
Other	4	1.1
How long has [your ex-partner] had control of your finances?		
Up to one year	6	1.7
1–4 years	76	21.7
5–10 years	114	32.6
More than 10 years	154	44.0
Is this control [by your ex-partner] still occurring?		
Yes	207	59.0
No	144	41.0
Do you feel that your ex-partner controls you through child support?		
Yes	273	79.8
No	60	17.5
[If controlled through child support], how controlled do you feel?		
A little	41	15.4
A lot	140	52.4
Completely	84	31.5
Has your ex-partner replaced physical abuse with economic abuse via child support as a way to control you now that you are separated?		
Yes	201	79.8
No	47	18.7
Have you experienced the deliberate withholding of child support?		
Yes	346	81.6
No	78	18.4
How long have you experienced deliberate child support withholding?		
Up to 1 year	102	29.7
1–4 years	107	31.2
5–10 years	80	23.3
More than 10 years	54	15.7
Are you still experiencing child support withholding?		
Yes	233	67.3
No	113	32.7
Have you experienced the deliberate minimization of your child support assessment?		
Yes	340	80.8
No	81	19.2
How long have you experienced deliberate child support assessment minimization?		
Up to one year	53	15.6
1–4 years	131	38.5
5–10 years	99	29.1
More than 10 years	57	16.8
Are you still experiencing child support assessment minimization?		
Yes	279	82.3
No	60	17.7

### The Nature of Child Support Financial Abuse

With specific respect to child support, 79.8% of respondents reported that their ex-partner controlled them through these payments, with the same proportion (79.8%) reporting that their ex-partner had replaced physical abuse with abuse via child support as a way to control them postseparation. For those women who reported feeling controlled through child support, 83.9% reported feeling controlled either “a lot” or “completely.”

**Figure 1.**
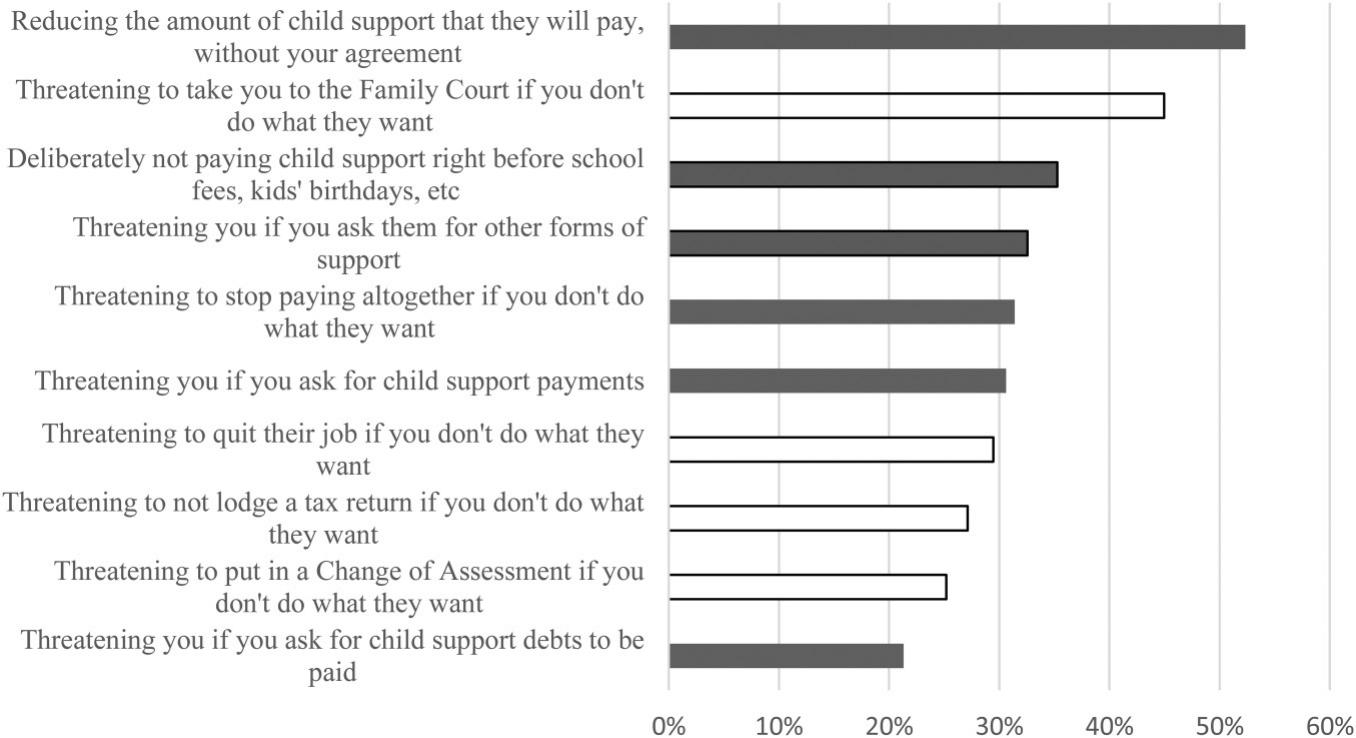
Top 10 most frequently reported ways that women's ex-partners used child support to control them.

When asked to choose from a list of 27 options regarding how their ex-partner controlled them through child support, the 258 women who provided responses selected an average of five controlling behaviors. The most frequently selected options (Figure 1) were, “Reducing the amount of child support that they will pay, without your agreement” (52.3%), “Threatening to take you to the family Court if you don't do what they want” (45.0%), and “Deliberately not paying child support right before school fees, kids’ birthdays, etc.” (35.3%). In Figure 1, lightly shaded items represent the minimization of child support liabilities, which would legally reduce the amount of child support owed, achieved through such actions as threatening to change child contact agreements, withholding income data, or reducing employment. Darkly shaded items represent nonpayment or coercive control over payments, such as withholding payments, threatening to stop or reduce payments, or threatening the mother if they sought payments.

### Child Support Payments as Tools for Perpetrating Financial Abuse

Women's experiences of child support were then compared to those of women who did not report abuse. Women who felt controlled by their ex-partner through child support appeared significantly more likely than those who did not feel controlled to answer “yes” to a question that asked whether their ex-partner was minimizing their child support assessment (Table 3).

**Table 3.** Minimization of Child Support Assessments as a Tactic of Financial Abuse.

		Minimization of child support assessments (%)	
		Yes	No
Controlled through child support	Yes	89.7	10.3
	No	56.7	43.3

ꭓ^2^(3) = 36.19, *p* < .001.

Almost nine out of every 10 women (89.7%) who felt financially controlled by their ex-partner through child support felt that their ex-partner had minimized the amount of child support owed. This proportion stood in contrast to only 56.7% of women who did not report experiencing financial control. With respect to child support nonpayment, women who reported that their ex-partner controlled them through child support appeared to be more likely to have received less child support than they expected in the previous month compared to women who were not controlled (Table 4).

**Table 4.** Child Support Payment Discrepancy Groups by Reported Control Through Child Support.

		Child support discrepancy groups (%)			
		Less child support than expected	Same child support as expected	More child support than expected	No child support expected or received
Controlled through child support	Yes	62.6	24.6	2.3	10.5
	No	26.5	55.9	5.9	11.8

ꭓ^2^(3) = 17.14, *p* < .001.

Nearly two-thirds (62.6%) of women who reported feeling controlled through child support received less money than they expected, compared to 26.5 for women who did not. By contrast, only 24.6% of women who felt controlled reported receiving the amount of child support that they expected, compared to 55.9% who did not feel controlled.

Women who reported being controlled by their ex-partner through child support expected to receive, on average, $716.78 (US$472) in child support in the previous month, and received an average of $242.25 (US$159), with an average shortfall of $453.65 (US$299, Table 5). Women who did not report being controlled, on average, expected $508.35 (US$335) and received $352.57 (US$232) in the previous month, with an average shortfall of $51.09 (US$34).

**Table 5.** Mean Amount of Child Support Expected and Received in the Previous Month, by Financial Control Group.

	Controlled through child support by ex-partner	*N*	*M* ($)
Amount of child support expected in the previous month	Yes	185	716.78
	No	34	508.35
Amount of child support received in the previous month	Yes	232	242.25
	No	51	352.57
Discrepancy between child support expected and received in the previous month^a^	Yes	170	−453.65
	No	33	−51.09

a *t*(−199.47) = −7.022, *p* < .001.

While not statistically significant, women whose ex-partner was using child support to control them expected to receive just over $200 more in child support in the previous month than those who did not feel controlled. Again, while not statistically significant, women who felt controlled through child support received just over $100 less child support than those who did not feel controlled. However, while the mean amount “expected” and “received” amounts of child support did not differ significantly between the groups, there was a significant difference in the mean child support underpayment for the previous month. Women who reported being controlled by their ex-partner experienced a significantly larger mean shortfall (−$453.65) in their child support payment, compared to those who did not (−$51.09).

## Discussion and Conclusion

In response to our research questions, we found that the proportion of single mothers in our study who had experienced financial control was striking, with almost 90% of respondents reporting such behavior. The high rate of women who had experienced financial control is indicative of the nature of the survey, which sought to understand separated women's financial safety, and to whom such a survey might appeal. However, while the results of our survey are not representative of all Australian separated mothers, the prevalence of financial abuse reflects the widespread nature of family violence reported by Australian single mothers (Summers, 2022). In addition, the rate of financial abuse that we reported was within the prevalence range reported by reported by other studies of economic abuse (Boxall & Morgan, 2021; Postmus et al., 2016).

On the role of the child support system in compounding the harms that low-income women experience through the manipulation of state-imposed debts, our findings provide several new insights. First, as child support commences soon after separation and continues until children reach adulthood, it was not surprising that the majority of women who reported being controlled by their ex-partner endured such behavior for many years. Similarly, given women's low incomes, the importance of child support to women's total income package, and the compounding impact of these payments on FTB payments (Cook, et al., 2023; DSS, 2023), it was also not surprising that over 80% of women reported that they felt significantly or completely controlled by their ex-partner through child support. However, the additional insights that our results provide beyond existing research were the ways through which such control was perpetrated and experienced over years and decades.

While previous research has identified court, taxation and child support processes as a significant sites of postseparation financial abuse (Douglas, 2021; Douglas & Nagesh, 2019; Kaye et al., 2021), no research to date has quantified the importance of these third-party institutions to women's experience of postseparation financial abuse. Our findings demonstrate the prevalence of third-party systems, such as the taxation system and the family court being used directly, or as threats, by perpetrators to ensure women's submission. While the top 10 most frequent ways that women were controlled through child support were each experienced by over a quarter of the sample, on average women experienced five such pathways. As such, it was the breadth and combination of systems used to enact financial abuse that was particularly insidious. The use of third-party systems to make threats or to enact financial harm makes the detection of financial abuse difficult, as seemingly innocuous actions within one system, such as making a late tax return, may be designed to enact financial harm within another. Reflecting on the challenge put forward within the National Plan (DSS, 2022b), there is a need for governments not only to build capacity to prevent and respond to financial abuse within individual institutions, but also across systems, such as child support, social security, taxation, and family law.

With respect to our third research question, our findings empirically demonstrate how the minimizing and withholding of child support are used as tactics of financial control. While previous Australian and international qualitative research has described these issues (Kaittila et al., 2022; Natalier, 2018; Sharp-Jeffs, 2021; Tegler et al., 2023) our findings add to these conceptual or qualitative accounts, by identifying statistically significant relationships that challenge conventional policy wisdom. In the Australian case, the finding that women experiencing financial control experienced greater discrepancies between the expected and received amounts of child support than their uncontrolled counterparts is of particular importance. Here, high child support liabilities have been taken as evidence of payers’ willingness to comply with the child support system, and thus presumably compliance measures (HRSCFCA, 2003; HRSCSPLA, 2015). However, our analysis has shown that while neither expected nor received payments were indicative on their own, it was the discrepancy between a relatively high value, but unpaid child support liability that was associated with financial abuse. This is particularly problematic because the government assumes 100% payment compliance for private collect agreements and reduces FTB on this basis. In the Australian case, the finding that women experiencing financial control experienced greater discrepancies between the expected and received amounts of child support than their uncontrolled counterparts is of particular importance. Here, high child support liabilities have been taken as evidence of payers’ willingness to comply with the child support system, and thus presumably compliance measures (HRSCFCA, 2003; HRSCSPLA, 2015). However, our analysis has shown that while neither expected nor received payments were indicative on their own, it was the discrepancy between a relatively high value, but unpaid child support liability that was associated with financial abuse. This is particularly problematic because the government assumes 100% payment compliance for private collect agreements and reduces FTB on this basis. A second, unquestioned assumption within the Australian child support system is that the timing of payments is unimportant. Previous qualitative research has raised this issue (Cook et al., 2015), but it has not been taken up in policy evaluations, analyses, or reform (DSS, 2022a). Rather, government policy has moved in the opposite direction, as amendments made in 2015 imposed vigorous collection methods for FTB “overpayments.” Parents who received child support back payments or retrospective child support liability calculations on the basis of their ex-partner's retrospectively lodged tax returns, particularly those collecting privately, could face retrospective FTB calculations and thus overpayment notices. Perpetrators’ ability to manipulate child support liabilities and payments—and the double penalty faced by women of reduced family payments and the prospect of FTB debts owed to the state—allows child support to be used as a financial weapon.

Manipulating the reliability of payments was also revealed as a tactic of financial abuse through the significantly larger discrepancies in child support payments experienced by women who felt controlled by their ex-partner. In addition, withholding payments before significant events was a common tactic. The fact that our survey was conducted over the Christmas and Australian summer school holiday period may have contributed to over a third of women indicating that payments had been withheld before significant events requiring expenditure, such as on children's presents and school fees. Our results provide support for anecdotal reports that payments are withheld by fathers at these key times as a means of inflicting psychological and symbolic harm upon mothers. Analyses of administrative data are required to definitively determine the relationship between significant events in children's lives and fathers’ noncompliance; but in the Australian case, this would only apply to the 50% of the caseload for which payment data exists (i.e., where payments are transferred by the department).

It is the state's compulsory imposition of debts upon separated parents that renders child support open to being used as a tool in the perpetration of financial abuse. While child support systems hold with them inherent financial risks for both payers and recipients, there are opportunities to amend the Australian system to minimize the compounding harms that single mothers disproportionately experience as primary caregivers, heads of low-income households, and victims of family violence.

### Policy Interventions to Limit Opportunities for Financial Abuse

Our study reveals the way that child support payments are withheld, delayed, or minimized to inflict financial harm. While previous reviews of the child support system have excluded a specific focus on compliance (HRSCFCA, 2003; HRSCSPLA, 2015), there are several practical policy solutions that could improve compliance and reduce the incidence of financial abuse. These reforms would take up the sentiment of the National Plan to End Violence Against Women and Children (DSS, 2022b) by strengthening the capacity of the Department responsible for FTB and child support as well as the Australian Taxation Office. The suggested reforms would prevent abuses of child support, FTB, and tax processes and ultimately reduce opportunities for the perpetration of postseparation financial abuse.

First, rather than promoting private collections, whereby payment outcomes are the responsibility of recipients, child support could be returned to a system of compulsory payments, facilitated through wage withholding or the tax system, as was a feature of the original Australian scheme (Alexander, 1995). To ensure compliance, more responsibility should be taken by the state to withhold payments at their source, rather than relying on potentially abusive parents to privately transfer payments and for would-be recipients to police these transactions. Such a system should remove the burden placed on recipients to monitor and enforce payments and could include penalties for payers who deliberately evade their responsibilities, such as by impacting on their credit ratings.

A second policy solution that would greatly reduce opportunities for financial abuse is to decouple child support from FTB so that recipients can rely on these valuable government payments even when child support payments are unreliable. This solution mirrors the recommendation of numerous policy review processes, including the government-initiated Women's Economic Equality Taskforce (2023) and Interim Economic Inclusion Advisory Committee (2023).

Taken together, our recommendations provide tangible reforms that could address the sentiment expressed by the Minister for Women and federal Treasurer in the 2022–23 Women's Budget Statement (Gallagher & Chalmers, 2023, p. 22) where they noted:

Where tax returns are delayed or not lodged by an absentee child support provider, it may affect calculations of parents’ entitlements, including accuracy of Family Tax Benefit payments, and reduce Services Australia's ability to intercept tax refunds to meet outstanding child support debts.

This can effectively see child support payments withheld and, in some circumstances, former partners may do this intentionally to inflict financial abuse on women. Our incremental policy recommendations provide means of buffering low-income victims of family violence from the compounding harms of the child support that are enacted by their ex-partner's manipulation of state-imposed debt. However, these recommendations leave the inherently harmful system intact. As has been argued elsewhere (Cook et al., 2023), a better solution—that would free all parents from the pernicious burden of state-imposed debts—would be to disband the child support system in place of a state-funded living wage.

In conclusion, our findings have demonstrated that the Australian child support system is a vehicle for financial abuse, with perpetration often structured and facilitated by state requirements and processes. Contrary to the assumption that violence ends once women leave physically violent partners (Summers, 2022), our survey revealed that single mothers continue to experience family violence in the form of financial abuse, long after separation. While the prevalence, experience, and tactics of financial abuse are alarming, there are practical reforms that the government can make that would significantly reduce the harms being perpetrated through the Australian child support scheme, but the ultimate—albeit far less politically palatable solution—may lie in dismantling the system that allows such debts to be weaponized.

## References

[bibr1-10778012241280057] Alexander L. (1995). Australia’s child support scheme: Much promised, little delivered? Family Matters 42(Spring/Summer), 6–11. 10.3316/agispt.19960104

[bibr2-10778012241280057] Australian Bureau of Statistics (ABS). (2022). Average weekly earnings, Australia. Australian Bureau of Statistics. http:////www.abs.gov.au/statistics/labour/earnings-and-working-conditions/average-weekly-earnings-australia/latest-release

[bibr3-10778012241280057] Australian Government. (2023). Women’s budget statement (Budget 2023–24). Commonwealth of Australia. http://budget.gov.au/content/womensstatement/download/womens_budget_ statement_2023-24.pdf

[bibr4-10778012241280057] Australian Law Reform Commission (2012). Family violence and commonwealth laws—improving legal frameworks (ALRC Report 117). Australian Government.

[bibr5-10778012241280057] Boxall H. Morgan A. (2021). Intimate partner violence during the COVID-19 pandemic: A survey of women in Australia (Special reports no.11). Australian Institute of Criminology. https://www.aic.gov.au/publications/special/special-11

[bibr6-10778012241280057] Chaudhri R. (2020). Tackling financial abuse with the doctrine of undue influence. Australian Journal of Family Law, 33(3), 191–212. 10.3316/agispt.20201102038987

[bibr7-10778012241280057] Cook K. (2013). Child support compliance and tax return non-filing: A feminist analysis. Australian Review of Public Affairs: Journal, 11(2), 43–64. 10.3316/family.a147025

[bibr8-10778012241280057] Cook K. (2019). The devaluing and disciplining of single mothers in Australian child support policy. In Australian mothering: Historical and sociological perspectives (pp. 381–402). Palgrave Macmillan. https://doi.org/10.1007/978-3-030-20267-5_18

[bibr9-10778012241280057] Cook K. Byrt A. Burgin R. Edwards T. Coen A. Dimopoulos G. (2023). *Financial abuse: the weaponisation of child support in Australia*. 10.26185/72dy-m137

[bibr10-10778012241280057] Cook K. Davis E. Davies B. (2008). Discrepancy between expected and actual child support payments: Predicting the health and health-related quality of life of children living in low-income, single-parent families. Child: Care, Health and Development, 34(2), 267–275. 10.1111/j.1365-2214.2007.00802.x 18257796

[bibr11-10778012241280057] Cook K. McKenzie H. Natalier K. (2015). Mothers’ experiences of child support: Qualitative research and opportunities for policy insight. Journal of Family Studies, 21(1), 57–71. 10.1080/13229400.2015.1011769

[bibr12-10778012241280057] Cook K. Skinner C. (2021). Technical fixes as challenges to state legitimacy: Australian separated fathers’ suggestions for child support policy reform. Social Politics: International Studies in Gender, State & Society, 28(2), 501–520. 10.1093/sp/jxz051

[bibr13-10778012241280057] Cozzolino E. (2018). Public assistance, relationship context, and jail for child support debt. Socius, 4, 1–25. 10.1177/2378023118757124

[bibr14-10778012241280057] Cozzolino E. Williams C. L. (2017). Child support queens and disappointing dads: Gender and child support compliance. Social Currents, 4(3), 228–245. 10.1177/2329496516663224

[bibr15-10778012241280057] Davidson P. Bradbury B. Wong M. (2020). Poverty in Australia 2020, Part 2: Who is affected? (ACOSS/UNSW Poverty and Inequality Partnership Report No. 4). Australian Council of Social Service [ACOSS]*.* http://povertyandinequality.acoss.org.au/wp-content/uploads/2020/05/Poverty-in-Australia-2020-Part-2-–-Who-is-affected_Final.pdf

[bibr16-10778012241280057] Department of Social Services. (2022a). *Services Australia child support extract data 2022. Child support program fact sheet – September quarter 2022*. https://data.gov.au/data/dataset/6379b974-e547-4303-a361-6edebbb52550/resource/6b8cb72e-3fff-4a23-b1e1-61123518132d/download/child-support-a3-factsheet-september-qtr-2022-finalr1-pdf-09.12.22.pdf

[bibr17-10778012241280057] Department of Social Services. (2022b). *National plan to end violence against women and children 2022-2032*. https://www.dss.gov.au/sites/default/files/documents/10_2022 /national_plan_accessible_version_for_website.pdf

[bibr18-10778012241280057] Department of Social Services (2023). *3.1.5.55 Reasonable maintenance action completed – private collect. In Family Assistance Guide (version 1.245)*. https://guides.dss.gov.au/family-assistance-guide/3/1/5/55

[bibr19-10778012241280057] de Vaus D. Gray M. Qu L. Stanton D. (2017). The economic consequences of divorce in six OECD countries. Australian Journal of Social Issues, 52(2), 180–199. 10.1002/ajs4.13

[bibr20-10778012241280057] Diduck A. (1995). The unmodified family: The child support act and the construction of legal subjects. Journal of Law and Society, 22(4), 527–548. 10.2307/1410613

[bibr21-10778012241280057] Douglas H. (2021). Women, intimate partner violence, and the law. Oxford University Press. 10.1093/oso/9780190071783.001.0001

[bibr22-10778012241280057] Douglas H. Nagesh R. (2019). Domestic and family violence, child support and ‘the exemption’. Journal of Family Studies, 27(4), 1–16. 10.1080/13229400.2019.1653952

[bibr23-10778012241280057] Eekelaar J. (1991a). Parental responsibility: State of nature or nature of the state? Journal of Social Welfare and Family Law, 13(1), 37–50. 10.1080/09649069108413929

[bibr24-10778012241280057] Eekelaar J. (1991b). Are parents morally obliged to care for their children? Oxford Journal of Legal Studies, 11(3), 340–353. 10.1163/9789004637702_007

[bibr25-10778012241280057] Fitzpatrick C. (2022). *Designed to disrupt: Reimagining banking products to improve financial safety.* Centre for Women’s Economic Safety. http://cwes.org.au/wp-content/uploads /2022/11/CWES_DesigntoDisrupt_1_Banking.pdf

[bibr26-10778012241280057] Gallagher K. Chalmers J. (2023). Budget 2023-24 women's budget statement. Commonwealth of Australia.

[bibr27-10778012241280057] Henriques-Gomes L. (2021, May 13). ‘Demeaning’ crackdown on single mothers scrapped, budget papers reveal. *The Guardian*. http://www.theguardian.com/australia-news/2021/may/13/demeaning-crackdown-on-single-mothers-scrapped-budget-papers-reveal

[bibr28-10778012241280057] House of Representatives Standing Committee Family and Community Affairs (2003). Every picture tells a story: Inquiry into child custody arrangements in the event of family separation. Commonwealth of Australia.

[bibr29-10778012241280057] House of Representatives Standing Committee on Social Policy and Legal Affairs (2015). From conflict to cooperation: Inquiry into the child support program. Commonwealth of Australia.

[bibr30-10778012241280057] House of Representatives Standing Committee on Social Policy and Legal Affairs (2021). Inquiry into family, domestic and sexual violence. Parliament of Australia.

[bibr31-10778012241280057] Interim Economic Inclusion Advisory Committee (2023). 2023–24 report to the Australian Government. Commonwealth of Australia. https://www.dss.gov.au/sites/default/files/documents/06_2023/eiac_report_8.06.23_0.pdf

[bibr32-10778012241280057] Johnson L. Chen Y. Stylianou A. Arnold A. (2022). Examining the impact of economic abuse on survivors of intimate partner violence: A scoping review. BMC Public Health, 22(1), 1–19. 10.1186/s12889-022-13297-4 35590302 PMC9121607

[bibr33-10778012241280057] Joint Select Committee on Australia’s Family Law System (2021a). Third interim report: Australia’s child support scheme. Commonwealth of Australia.

[bibr34-10778012241280057] Joint Select Committee on Australia’s Family Law System (2021b). Final report. Commonwealth of Australia.

[bibr35-10778012241280057] Kaittila A. Hakovirta M. Kainulainen H. (2022). Types of economic abuse in postseparation lives of women experiencing IPV: A qualitative study from Finland. Violence Against Women, 30(2), 426–444. 10.1177/10778012221127727 36177605 PMC10775644

[bibr36-10778012241280057] Kaye M. Booth T. Wangmann J. (2021). Compromised ‘consent’ in Australian Family Law Proceedings. International Journal of Law, Policy and Family, 35(1), 1–25. 10.1093/lawfam/ebab033

[bibr37-10778012241280057] Knaus C. (2018, April 4). Welfare crackdown on relationships a ‘double standard’ not applied to MPs. The Guardian. https://www.theguardian.com/australia-news/2018/apr/04/welfare-crackdown-on-relationships-a-double-standard-not-applied-to-mps

[bibr38-10778012241280057] Natalier K. (2012). Descriptions of loss and resilience among fathers paying child support. Journal of Family Studies, 18 *(*2-3), 246–255. 10.5172/jfs.2012.2477

[bibr39-10778012241280057] Natalier K. (2018). State facilitated economic abuse: A structural analysis of men deliberately withholding child support. Feminist Legal Studies, 26(2), 121–140. 10.1007/s10691-018-9376-1

[bibr40-10778012241280057] Natalier K. Hewitt B. (2010). ‘It’s not just about the money’: Non-resident fathers’ perspectives on paying child support. Sociology, 44(3), 489–505. 10.1177/0038038510362470

[bibr41-10778012241280057] Oldham T. Smyth B. (2018). Child support compliance in the USA and Australia: To persuade or punish? Family Law Quarterly, 52(2), 1–17. 10.3316/family.a159886

[bibr42-10778012241280057] PenzeyMoog E. Slakoff D. C. (2021). As technology evolves, so does domestic violence: Modern-day tech abuse and possible solutions. In Bailey J. Flynn A. Henry N. (Eds.), The emerald international handbook of technology-facilitated violence and abuse (pp. 643–662). Emerald Publishing Limited. 10.1108/978-1-83982-848-520211047

[bibr43-10778012241280057] Postmus J. L. Hoge G. L. Breckenridge J. Sharps-Jeff N. Chung D. (2020). Economic abuse as an invisible form of domestic violence: A multicountry review. Trauma, Violence, and Abuse, 21(2), 261–283. 10.1177/152483801876416 29587598

[bibr44-10778012241280057] Postmus J. L. Plummer S. McMahon S. Murshid N. S. Kim M. S. (2012). Understanding economic abuse in the lives of survivors. Journal of Interpersonal Violence, 27(3), 411–430. 10.1177/08862605114216 21987509

[bibr45-10778012241280057] Postmus J. L. Plummer S. B. Stylianou A. M. (2016). Measuring economic abuse in the lives of survivors: Revising the scale of economic abuse. Violence Against Women, 22(6), 692–703. 10.1177/1077801215610012 26508530

[bibr46-10778012241280057] Rinta-Kahila T. Someh I. Gillespie N. Indulska M. Gregor S. (2023). Managing unintended consequences of algorithmic decision-making: The case of Robodebt. Journal of Information Technology Teaching Cases, 14(1), 165–171. 10.1177/20438869231165538

[bibr47-10778012241280057] Rush M. (2015). Between two world of father politics: USA or Sweden? Manchester University Press. 10.7228/manchester/9780719091896.001.0001

[bibr48-10778012241280057] Scott A. (2023). Post-separation financial abuse, the money taboo and the family justice system: Perspectives from Aotearoa New Zealand. In Maclean M. Treloar R. (Eds), Research handbook on family justice system (pp. 176–194). Edgar Elgar. 10.4337/9781800881402.00020

[bibr49-10778012241280057] Services Australia (2023). Basic Formula. Australia: Australian Government. Available at: https://www.servicesaustralia.gov.au/basic-child-support-formula?context=21911

[bibr50-10778012241280057] Sharp-Jeffs N. (2021). Understanding the economics of abuse: An assessment of the economic abuse definition within the domestic abuse bill. Journal of Gender-Based Violence, 5(1), 163–173. 10.1332/239788220X16076181041680

[bibr51-10778012241280057] Shephard A. (2005). The Australian child support agency: Debt study and follow-up on intensive debt collection processes. Family Court Review, 43(3), 387–401. 10.1111/j.1744-1617.2005.00041.x

[bibr52-10778012241280057] Skinner C. Meyer D. R. Cook K. Fletcher M. (2017). Child maintenance and social security interactions: The poverty reduction effects in model lone parent families across four countries. Journal of Social Policy, 46(3), 495–516. 10.1017/S0047279416000763

[bibr53-10778012241280057] Sleep L. (2019). Domestic violence, social security and the couple rule. ANROWS. http://d2rn9gno7zhxqg.cloudfront.net/wp-content/uploads/2019/07/18032453/RP.17.02 _Sleep_D_RR_social-security_couple-rule.pdf

[bibr54-10778012241280057] Summers A. (2022). The choice: Violence or poverty. Labour and Industry, 32(4), 349–357. 10.26195/3s1r-4977

[bibr55-10778012241280057] Tegler H. Fernqvist S. Flinkfeldt M. (2023). “And all hell broke loose”: How child maintenance regulations contribute to Re-actualizing intimate partner violence between separated parents in Sweden. Journal of Family Violence, 38, 127–138. 10.1007/s10896-022-00365-x

[bibr56-10778012241280057] Tejani S. (June Quarter, 2022). Poverty Lines: Australia. Melbourne Institute of Applied Economic and Social Research. https://melbourneinstitute.unimelb.edu.au/__ data/assets/pdf_file/0008/4288661/Poverty-Lines-Australia-June-2022.pdf#:∼:text = The%20Melbourne%20Institute%20of%20Applied%20Economic%20and%20Social,of%20whom%20is%20working%2C%20and%20two%20dependent%20children

[bibr57-10778012241280057] Vnuk M. (2010). Merged or omitted? What we know (or don’t) about separated mothers who pay or should pay child support in Australia. Journal of Family Studies, 16(1), 62–76. 10.5172/jfs.16.1.62

[bibr58-10778012241280057] Vnuk M. (2019). Keeping mum: Characteristics and family dynamics of mothers who are liable to pay child support in Australia. Australian and New Zealand Journal of Family Therapy, 40(1), 127–142. 10.1002/anzf.1348

[bibr59-10778012241280057] Whiteford P. (2021). Debt by design: The anatomy of a social policy fiasco – or was it something worse? Australian Journal of Public Administration, 80(2), 340–360. 10.1111/1467-8500.12479 .

[bibr60-10778012241280057] Women’s Economic Equality Taskforce. (2023). Women’s economic equality taskforce final report. Commonwealth of Australia. https://www.pmc.gov.au/sites/default/files/resource/download/womens-economic-equality-taskforce-final-report.pdf

[bibr61-10778012241280057] Women’s Legal Services Australia. (2024). Non-Payment of Child Support as Economic Abuse of Women and Children: A Literature Review. https://www.wlsa.org.au/wp-content/uploads/2024/05/Womens-Legal-Services-Australia-Child-Support-Literature-Review-May-2024.pdf

